# A comparative evaluation of antibiotic and synbiotic supplementation on production performance and necrotic enteritis severity in broilers during an experimental necrotic enteritis challenge

**DOI:** 10.3389/fphys.2024.1511380

**Published:** 2025-01-15

**Authors:** Bikas Raj Shah, Walid Ghazi Al Hakeem, Revathi Shanmugasundaram, Ramesh K. Selvaraj

**Affiliations:** ^1^ Department of Poultry Science, University of Georgia, Athens, GA, United States; ^2^ Toxicology and Mycotoxin Research Unit, Agriculture Research Service, United States Department of Agriculture, Athens, GA, United States

**Keywords:** AGP, antibiotic, immune response, necrotic enteritis, performance, synbiotic, Broilers

## Abstract

The ban on antibiotics in the poultry diet resulted in re-emergence of several infectious diseases including necrotic enteritis (NE). These infectious diseases are leading to poor health and welfare as well as production and economic loss. Synbiotic could be a potential candidate to replace the antibiotics in poultry diet. Therefore, a 35-day study was conducted to compare the efficacy of synbiotic (PoultryStar^®^ME) and antibiotic (Stafac^®^50, Virginiamycin) supplementation during an experimentally induced necrotic enteritis infection. A total of 360 day-old chicks were randomly assigned to four treatment groups: Antibiotic, Challenge + Antibiotic, Synbiotic, and Challenge + Synbiotic, each with 6 replicates. The treatment groups referred as “Challenge + Antibiotic” and “Challenge + Synbiotic” were challenged, while their respective non-challenged treatment groups were “antibiotic” and “synbiotic”. NE in birds was induced by gavaging 1 × 10^4^ oocysts of *Eimeria maxima* on day 14 **(D14)** and 1 × 10^8^ CFU/mL of *Clostridium perfringens* on D19, 20, and 21. Both synbiotic and antibiotic supplementation during the NE challenge did not improve BW gain, feed intake, and feed conversion ratio at the end of the experiment (D0-35). However, antibiotic supplementation reduced mortality during the week of the challenge (D14-21) *(P < 0.001)*. At D21, both synbiotic and antibiotic supplementation during the NE challenge did not decrease the intestinal lesion score *(P < 0.001)* compared to their respective non-challenged treatment groups. At D21, synbiotic supplementation during the NE challenge did not decrease intestinal permeability *(P = 0.04)* compared to the synbiotic group. At D21, antibiotic supplementation during the NE challenge increased the CD4+:CD8+ T cells *(P < 0.001)* in the cecal tonsil. It can be concluded that synbiotic supplementation elicited an immune response, decreasing the inflammatory response in the intestine and ameliorating the NE infection. Therefore, synbiotic could be a potential alternative to replace antibiotics in the poultry industry, but their efficacy needs to be improved through blending additional probiotics and prebiotics, and further exploration is required.

## 1 Introduction

Necrotic enteritis **(NE)** is an infectious disease in poultry which is characterized by depression, reluctance to move, ruffled feathers, diarrhea, loss of appetite, and anorexia (Ficken, 1991). The etiological agent of NE is *Clostridium perfringens*, an anaerobic, spore-forming, gram-positive bacterium (Porter, 1998). Usually, there are five toxinotypes of *C. perfringens* (A-E), producing at least 21 toxins or potentially toxic exo-proteins (Brynestad and Granum, 2002). However, a study suggested that the necrotic enteritis type B **(netB)** toxin, produced by *C. perfringens* type G, is responsible for the etiology of NE (Keyburn et al., 2008). Additionally, coccidial infection caused by *Eimeria brunetti* or *E. maxima* has also been found to be associated with the induction of NE (Helmboldt and Bryant, 1971). Several other factors predispose to NE infection, including dietary factors, stress, stocking density, dysbiosis, and changes in immunological and physiological aspects of the intestine (Adhikari et al., 2020). While *C. perfringens* is commensal to poultry intestine (Lu et al., 2003), mild infection is insufficient to produce the disease. Predisposing factors play a critical role in the development of NE infection by damaging intestinal epithelium, increasing mucus production, and altering food transit time in gut (Moore, 2016).

Previously, antimicrobial growth promoters **(AGPs)** were used to control the incidence of NE and other infectious diseases in poultry. However, the overuse of AGPs in poultry diets resulted in increased bacterial resistance and the presence of antimicrobial residues in animal product as well as ultimately in the environment. Consequently, AGPs have been banned in poultry diets, necessitating an immediate alternative with similar infection controlling properties (Dibner and Richards, 2005). A potential alternative to AGPs could be a synbiotic, which is defined as the combination of probiotics and prebiotics, that interacts synergistically to benefit the host (Schrezenmeir and de Vrese, 2001). Synbiotic supplementation emerges as a promising alternative to AGPs, as it has been shown to ameliorate sub-clinical NE infections in poultry through improved production performance, decreased *C. perfringens* load in ceca, immunomodulation, and improved mucosal immune response (Shanmugasundaram et al., 2020a).

Several mechanisms regarding functions of synbiotic are proposed in the literature, as synbiotic supplementation can provide a prebiotic (food for probiotic bacteria), that can aid probiotic bacteria in proliferating and protecting the gut against pathogenic bacteria (Shah et al., 2023; Shanmugasundaram et al., 2020b). Additionally, synbiotic supplementation can enhance the gut barrier function by enhancing the tight junction’s integrity (Villagrán-de la Mora et al., 2019). Synbiotic supplementation can also decrease gut inflammation by modulating cytokine production and promoting anti-inflammatory pathways (Song et al., 2022). This leads to an improvement in nutrient utilization and production performance of broilers.

Numerous studies are being conducted to discover an alternative to AGPs, among which synbiotic is emerging as a supplement of interest due to their synergistic effects. However, very few studies have compared the efficacy of synbiotic when supplemented in feed from day 0 of age, similar to AGPs. Therefore, we hypothesized that synbiotic supplementation form day 0 of age will provide better protection and faster recovery during experimentally induced NE challenge compared to AGP. This study aims to investigate and compare the effects of synbiotic and AGP supplementation on production performance, intestinal integrity, mucosal humoral immune response, and mucosal cell-mediated immune response in broiler birds during *C. perfringens*-induced experimental NE infection.

## 2 Materials and methods

All animal protocols were approved by the Institutional Animal Care and Use Committee **(IACUC)** at the University of Georgia (IACUC protocol # A2021 06-002-A5). Researchers involved in this study received training from the University of Georgia in animal care and handling. Throughout the *in-vivo* experiment, the researchers monitored the birds for any signs of sickness at least twice a day. In case of any adverse event, euthanasia was performed through cervical dislocation.

### 2.1 Birds, diets, and NE infection

A total of 360 day-old chicks were randomly assigned to four treatment groups: Antibiotic **(A)**, Challenge + Antibiotic **(C + A)**, Synbiotic **(S)**, and Challenge + Synbiotic **(C + S)**. Among these groups, C + A and C + S are challenged treatment groups, while A and S are their respective non-challenged treatment groups. Each treatment has six replicate pens (n = 6) with 15 birds per pen. All birds in the treatment groups were fed with corn and soybean meal-based basal diet (Table 1) meeting the minimum requirements outlined by the National Research Council (NRC, 1994). The diet of birds in S and C + S treatment groups was supplemented with synbiotic (0.5 g/kg) (PoultryStar^®^ME, Biomin America, Inc.) from D0. This synbiotic included 2 × 10^11^ CFU/kg of four live strains of probiotic bacteria from the adult chickens (*L*. *reuteri*, *E*. *faecium*, *B*. *animalis*, and *P*. *acidilactici*) along with prebiotic, Fructooligosaccharide **(FOS)**. Similarly, the diet of birds in A and C + A treatment groups was supplemented with antibiotic (0.18 g/kg) (Stafac^®^50, Virginiamycin) from D0. The birds in the C + S and C + A treatment groups were orally gavaged with 1 × 10^4^ *E. maxima* oocysts on D14, followed by 1 × 10^8^ CFU/mL of *C. perfringens* (CP6) on D19, D20, and D21, to experimentally induce NE infection. Conversely, non-challenged birds in S and A treatment groups were orally gavaged with 1 mL of 1X PBS on above mentioned time points. On D21, D28, and D35, one bird from each pen was euthanized through cervical dislocation, and samples were collected for further analysis.

**TABLE 1:** Basal diets; Starter and Finisher with ingredients and nutrient composition.

Ingredients	Basal diet	
	Starter (D0-D14) %	Finisher (D15-D35) %
Corn	55.25	64.35
Soybean meal	38.18	29.29
Soybean oil	2.07	2.5
Dical	1.62	1.32
Limestone	1.34	1.17
NaCl	0.43	0.38
D.L. Methionine	0.42	0.4
L Lysine	0.26	0.19
Choline chloride	0.25	0.22
Vitamins premix^a^	0.1	0.1
Trace mineral premix^b^	0.08	0.08
Total	100	100
Calculated nutrient composition		
ME, kcal/kg	3,050	3,168
Crude protein, %	21.44	18.94
Crude fat, %	4.55	4.05
Lysine, %	1.31	1.05
Calcium, %	0.95	0.76
TSAA, %	0.91	0.82
Threonine, %	0.87	0.71
Methionine, %	0.56	0.55
Available phosphorus, %	0.45	0.38

^a^ Vitamin mix provided the following per kg of diet: 2.4 mg thiamin-mononitrate, 44 mg nicotinic acid, 4.4 mg riboflavin, 12 mg D-Ca pantothenate, 12 g vitamin B12, 2.7 mg pyridoxine-HCl, 0.11 mg D-biotin, 0.55 mg folic acid, 3.34 mg menadione sodium bisulfate complex, 220 mg choline chloride, 1,100 IU, cholecalciferol, 2,500 IU, trans-reinyl acetate, 11 IU, all-rac-tocopherol acetate, and 150 mg ethoxyquin.

^b^ Trace mineral mix provided the following per kg of diet: 101 mg MnSO_4_.H_2_O, 20 mg FeSO_4_.7H_2_O, 80 mg Zn, 3 mg CuSO_4_.5H_2_O, 0.75 mg ethylene diamine dihydroiodide, 20 mg MgO, and 0.3 mg sodium selenite.

### 2.2 Intestinal permeability

On D21, D28, and D35 of the study, one bird from each pen was orally gavaged with 2.2 mg/mL of fluorescein isothiocyanate-dextran (**FITC-d**; 100 mg, MW 4,000; Sigma-Aldrich, Canada). Two hours later, the birds were euthanized, and blood was collected from the heart and placed in opaque tubes. After couple of hours serum was separated and 100 μL of serum from each bird was added in duplicates to a 96-well black plate. The concentration of FITC-d in the blood serum was measured using a microplate reader at a wavelength of 485 nm and an emission wavelength of 528 nm. The concentration of FITC-d/mL in blood serum was calculated using a standard curve, where a higher concentration of FITC-d indicates greater gut permeability.

### 2.3 Mid-gut lesion score

On D21 of the study, three birds from each pen were randomly selected, sacrificed, and their mid-gut was inspected for any visible necrotic lesions. Lesion scoring was recorded on a scale of 0–3, where 0 indicates normal intestine and 3 indicates most severe condition of the intestine. Specifically, a score of 1 indicates thin-walled or friable lesions, 2 indicates focal necrosis or ulceration, and 3 indicates large patches of necrosis (Zhang et al., 2010).

### 2.4 CD4^+^ and CD8^+^ T Cells isolation and flowcytometry

On D21, D28, and D35 of the study, one cecal tonsil **(CT)** and half of the spleen were collected from one bird per pen in a 5 mL tube containing 3 mL of incomplete RPMI-1640 medium. These samples were then stored on ice and transported to the laboratory. In this experiment, sample preparation and flowcytometry was conducted following previously described method (Shanmugasundaram and Selvaraj, 2012a) with minor modifications. Cecal tonsil was cut longitudinally and placed on a cell strainer (Catalog number 431750, Corning, New York, NY) with mucosa layer facing downwards. Similarly, section of spleen was also placed on a cell strainer. Then tissue section was gently crushed using syringe plunger using incomplete RPMI-1640 to obtain a single cell suspension. Single cell suspension was centrifuged to obtain a cell pellet by discarding the supernatant. Cell pellet was resuspended in incomplete RPMI-1640, and cell was counted using trypan blue exclusion method. Approximately 1 × 10^6^ cells were mixed with FITC conjugated mouse anti-chicken CD4 cells (Catalog number 8210-90, Southern Biotech, Birmingham, AL) at a 1:250 dilution, or FITC conjugated mouse anti-chicken CD8 cells (Catalog number 8220-02, Southern Biotech) at a 1:450 dilution, along with unlabeled mouse IgG at a 1:100 dilution. The cell cocktails were incubated for 20 min at 4°C. Unbound primary antibodies were eliminated by centrifuging at 400 xg at 10°C for 5 minutes. The percentages of CD4^+^ and CD8^+^ T cells were determined for both the CT and spleen by using the 96-well plates in the flow cytometer (Guava EasyCyte, Millipore, Billerica, MA). The CD4^+^ and CD8^+^ T cell percentages were measured by gating cells based on the forward-scatter and side-scatter plot for lymphocytes, and the CD4+:CD8+ T cell ratio was calculated.

### 2.5 Quantification of anti *C. perfringens* IgA in the bile

On D21, D28, and D35 of the study, bile was aseptically collected from one bird per pen in a 2 mL Eppendorf tube, kept in ice, and then transported to the laboratory. In this experiment, ELISA was conducted following previously described methods (Shanmugasundaram et al., 2020a) with minor modifications. The pure culture of *C. perfringens* underwent six successive freeze-thaw-lyse cycles to produce the *C. perfringens* protein, which will act as antigen for coating ELISA plate. Glass beads of size 425-600 mm in diameter were used in a tissue lyser to mechanically lyse the culture for 5 min at 50 Hz. 10 mg/mL of *C. perfringens* protein, diluted in 0.1M carbonate buffer (pH 9.6) was coated to a 96-well ELISA plate and incubated overnight at 4°C. The plate was washed the next day, and the coating was blocked using 1X PBS containing 8% non-fat dry milk and a 0.05% tween. Bile samples were diluted in 1X PBS containing 8% non-fat dry milk and 0.05% tween at 1:800 and then added to the wells. Then, anti-chicken IgA conjugated with horseradish peroxidase was diluted in 1X PBS containing 5% non-fat dry milk and 0.05% tween at 1:100,000 and added to the wells. Furtherly, 3,3,5,5-tetramethylbenzidine substrate was added to the wells leading to a reaction causing color change, and the reaction was halted using 1N HCl. The OD values were determined using a microplate ELISA reader (BioTek, VT, United States) at 450 nm, and the anti *C. perfringens* IgA antibody levels were reported as mean OD values.

### 2.6 Expression of tight junction proteins in the jejunum and expression of cytokine genes in the cecal tonsil

On D21, D28, and D35 of the study, the jejunal section and a CT were aseptically collected and preserved in RNA later. After 5 days, the tissue sections were removed from the RNA later and stored at −20°C. Tri reagent was used to extract the total RNA from the tissue sections. A nanodrop spectrophotometer was used to assess the quantity and quality of RNA. The RNA was reverse transcribed into cDNA using oligodt primers and was then analyzed for gene expression of IL-10, Interferon-gamma **(IFN-γ)**, Transforming growth factor-beta **(TGF-β)**, Zonula occludens-1 **(ZO-1)**, and Claudin-1 **(CL-1)** by real-time rtPCR (CFX96 Touch Real-Time System, BioRad) using SyBr green after normalizing for ribosomal protein S13 **(RPS13)**. The relative fold change of target genes was calculated, as previously mentioned (Shanmugasundaram et al., 2019). Table 2 provides the primer sequences for the housekeeping gene and the target genes.

**TABLE 2:** Primer sequences of the housekeeping gene, cytokines, and tight junction proteins for real-time PCR.

Gene name	Primer sequence (5′-3′)	T_a_	References
RPS-13	F: CAAGAAGGCTGTTGCTGTICG	55.50°C	Hutsko et al. (2016)
	R: GGC​AGA​AGC​TGT​CGA​TGA​TT		
IL-10	F: GAG​GAG​CAA​AGC​CAT​CAA​GC	57.50°C	Shanmugasundaram et al. (2013)
	R: CTC​CTC​ATC​AGC​AGG​TAC​TCC		
TGF- β	F: CAG​AGC​ATT​GCC​AAG​AAG​C	59.00°C	Shanmugasundaram and Selvaraj (2012b)
	R: GCA​CGC​AGC​AGT​TCT​TCT​C		
IFN- γ	F: GTG​AAG​AAG​GTG​AAA​GAT​ATC​ATG​GA	57.00°C	Kaiser et al. (2000)
	R: GCT​TTG​CGC​TGG​ATT​CTC​A		
ZO-1	F: TGT​AGC​CAC​AGC​AAG​AGG​TG	55.00°C	Oxford and Selvaraj (2019)
	R: CTG​GAA​TGG​CTC​CTT​GTG​GT		
CL-1	F: CAT​ACT​CCT​GGG​TCT​GGT​TGG​T	57.50°C	Chen et al. (2017)
	R: GAC​AGC​CAT​CCG​CAT​CTT​CT		

Primer sequence: F, forward; R, reverse | T_a_, Annealing temperature

### 2.7 Statistical analysis

The data were analyzed using Jmp^®^ Pro 16 (JMP Statistical Discovery LLC) and figures were made using Prism 10 (GraphPad Software). Parametric data were analyzed through one-way ANOVA to identify differences between treatments followed by the Student’s T-test for mean comparison. Non-parametric data (lesion score) were analyzed using the Kruskal–Wallis test followed by the Dunn test for pairwise comparison. Each pen was considered an experimental unit. The level of significance was set at P < 0.05.

## 3 Results

### 3.1 Effect of synbiotic vs. antibiotic supplementation on production parameters and mortality

Till D14 of age (pre-challenge), no significant differences were observed in BW gain, feed intake **(FI)**, and feed conversion ratio **(FCR)** across all the treatment groups (Table 3).

**TABLE 3:** Effect of Synbiotic vs. Antibiotic Supplementation on Production Parameters and Mortality.

	Antibiotic	Challenge + antibiotic	Synbiotic	Challenge + synbiotic	SEM	*P-value*
Before challenge (0–14 days)						
BW gain (kg)	0.33	0.34	0.35	0.33	0.01	*0.68*
Feed intake (kg)	0.50	0.49	0.51	0.51	0.06	*0.26*
FCR	1.54	1.45	1.49	1.55	0.05	*0.44*
After Challenge (14–35 days)						
BW gain (kg)						
0–21 days	0.78^a^	0.66^b^	0.8^a^	0.65^b^	0.03	***<0.001***
0–28 days	1.46^a^	1.32 ^ab^	1.45^a^	1.20^b^	0.05	***<0.001***
0–35 days	2.03	1.96	2.06	1.91	0.06	*0.29*
Feed intake (kg)						
0–21 days	1.33	1.24	1.3	1.27	0.04	*0.42*
0–28 days	2.21	2.09	2.23	2.12	0.06	*0.26*
0–35 days	3.15	3.13	3.27	3.19	0.07	*0.56*
FCR						
0–21 days	1.75	1.87	1.64	1.99	0.09	*0.05*
0–28 days	1.53^b^	1.58^b^	1.54^b^	1.79^a^	0.06	***0.03***
0–35 days	1.56	1.6	1.59	1.68	0.04	*0.27*
Mortality (%)						
14–21 days	3.33^b^	7.78^b^	0.00^b^	35.56^a^	4.29	***<0.001***
21–28 days	0.00^b^	7.6^a^	0.00^b^	1.67^b^	1.63	***0.001***
28–35 days	0.00	0.00	0.00	0.00	-	*-*

Day-old chicks were distributed into four treatment groups. On days 14, 21, 28, and 35, average body weight and feed weight were recorded to evaluate production parameters. Mortality was recorded throughout the experiment and analyzed at D35. Values with no common superscript differ significantly (P < 0.05). Bold P-values indicates the significance.

At D21 of age, both synbiotic and antibiotic supplementation during the NE challenge did not result in increased BW gain (P < 0.001) in birds compared to their respective non-challenged treatment groups (Table 3). The C + S treatment group exhibited a 150 g lower BW gain, while the C + A treatment group showed a 120g lower BW gain when compared to the S and A treatment groups respectively. However, at D21 of age, no significant differences were observed in FI and FCR across all the treatment groups.

At D28 of age, synbiotic supplementation during the NE challenge did not result in increased BW gain (P < 0.001) in birds compared to the respective non-challenged treatment group. In contrast, antibiotic supplementation during the NE challenge resulted in comparable BW gain when compared to the respective non-challenged treatment group (Table 3). Birds in the C + S treatment group exhibited a 250 g lower BW gain compared to the S treatment group. However, no significant difference was observed in FI on D28 across all treatment groups. At D28 of age, synbiotic supplementation during the NE challenge did not decrease the FCR (P = 0.03) of birds compared to the respective non-challenged treatment group. On the other hand, antibiotic supplementation during the NE challenge showed a comparable FCR when compared to the respective non-challenged treatment group. Birds in the C + S treatment group had a 0.25 points higher FCR when compared to the S treatment group.

At D35 of age, no significant differences were observed in BW gain, FI, and FCR across all the treatment groups (Table 3).

During D14-21 of age (week of challenge), synbiotic supplementation did not result in a decrease in mortality, while antibiotic supplementation showed comparable mortality (P < 0.001) when compared to the respective non-challenged treatment group (Table 3). The percentage mortality of birds in the C + S treatment group was 35.56% higher when compared to the S treatment group. However, during D21-28 of age (1-week post challenge), synbiotic supplementation resulted in comparable mortality, while antibiotic supplementation did not decrease mortality (P = 0.00) compared to the respective non-challenged treatment groups. The percentage mortality of birds in the C + A treatment group was 7.6% higher when compared to the A treatment group. At D28-35 of age (two-weeks post challenge), no mortality was observed across all the treatment groups.

### 3.2 Effect of synbiotic vs. antibiotic supplementation on mid-gut lesion score

At D21 of age, both synbiotic and antibiotic supplementation during the NE challenge did not result in a significant decrease in the lesion scores (P < 0.001) in the mid-gut of birds when compared to their respective non-challenged treatment groups (Table 4). The birds in C + S and C + A treatment groups exhibited 36.19- and 25.81- points higher rank score mean when compared to S and A treatment groups respectively.

**TABLE 4:** Effect of Synbiotic vs. Antibiotic supplementation on mid-gut lesion score.

Treatments	Lesion score				Rank score means	n	ChiSq *P-value*
	0	1	2	3			
Antibiotic	18	0	0	0	21^b^	18	*< 0.001*
Challenge + Antibiotic	5	6	1	6	46.81^a^	18	
Synbiotic	18	0	0	0	21^b^	18	
Challenge + Synbiotic	0	6	5	7	57.19^a^	18	

Day-old chicks were distributed into four treatment groups. On day 21, 3 birds/pen were sacrificed, and the mid-gut was inspected for determining the lesion scores. Rank Score Means, and the difference in rank score means were calculated using the Wilcoxon/Kruskal–Wallis Test. Values with no common superscript differ significantly (P < 0.05).

### 3.3 Effect of synbiotic vs. antibiotic supplementation on intestinal permeability

**FIGURE 1:**
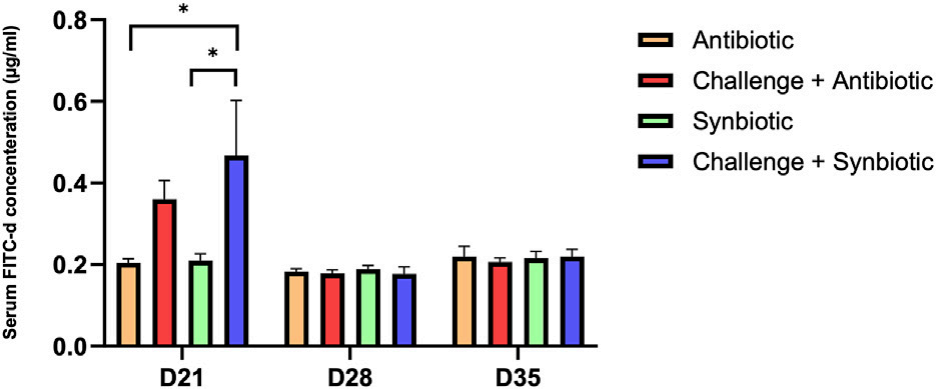
Effect of synbiotic vs. antibiotic supplementation on intestinal permeability of broiler birds under an experimental NE challenge. Day-old chicks were distributed into four treatment groups. At days 21, 28, and 35, gut permeability was measured by serum FITC-d assay. Bars connected with star/s differ significantly (*P < 0.05; **P < 0.01; ***P < 0.001; ****P < 0.0001). *D21: P-value = 0.04; D28: P-value = 0.85; D35: P-value = 0.94*.

At D21 of age, synbiotic supplementation during the NE challenge did not result in a significant reduction in intestinal permeability (P = 0.04) compared to the respective non-challenged treatment group. In contrast, antibiotic supplementation during the NE challenge showed comparable intestinal permeability compared to the respective non-challenged treatment group (Figure 1). Birds in the C + S treatment group exhibited a 0.26 mg/mL higher serum FITC-d concentration compared to the S treatment group.

At D28 and D35 of age, no significant differences were observed in serum FITC-d concentration across all the treatment groups.

### 3.4 Effect of synbiotic vs. antibiotic supplementation on CD4+:CD8+ T Cells in the Cccal Ttnsil

**FIGURE 2:**
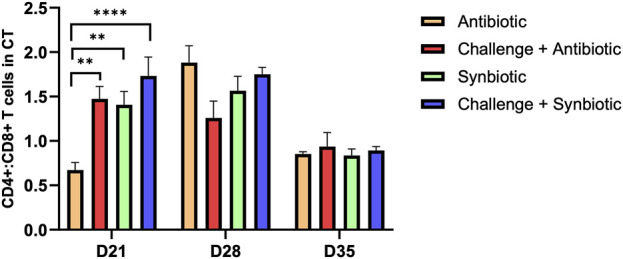
Effect of synbiotic vs. antibiotic supplementation on CD4+:CD8+ T cells in CT of broiler birds under an experimental NE challenge. Day-old chicks were distributed into four treatment groups. At days 21, 28, and 35, flowcytometry was performed to identify the percentage of CD4^+^ and CD8^+^ T cells in the cecal tonsil. Bars connected with star/s differ significantly (*P < 0.05; **P < 0.01; ***P < 0.001; ****P < 0.0001). *D21: P-value < 0.001; D28: P-value = 0.06; D35: P-value = 0.86*.

At D21 of age, synbiotic supplementation during the NE challenge resulted in comparable CD4+:CD8+ T cells in the CT compared to the respective non-challenged treatment group. Meanwhile, antibiotic supplementation during the NE challenge increased the CD4+:CD8+ T cells in the CT when compared to the respective non-challenge treatment group (Figure 2). Birds in the C + A treatment group exhibited a 0.8-point higher CD4+:CD8+ T cells in the CT compared to the A treatment group.

At D28 and D35 of age, no significant differences were observed in CD4+:CD8+ T cells in the CT of birds across all the treatment groups.

### 3.5 Effect of synbiotic vs. antibiotic supplementation on CD4+:CD8+ T Cells in the spleen

**FIGURE 3:**
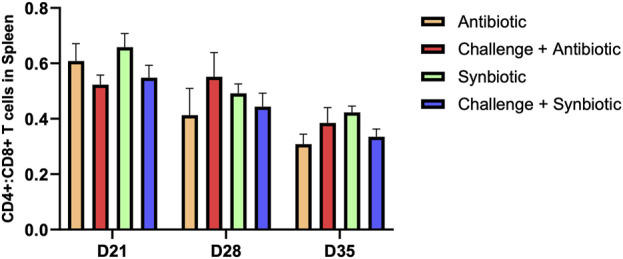
Effect of synbiotic vs. antibiotic supplementation on CD4+:CD8+ T cells in the spleen of broiler birds under an experimental NE challenge. Day-old chicks were distributed into four treatment groups. At days 21, 28, and 35, flowcytometry was performed to identify the percentage of CD4^+^ and CD8^+^ T cells in the spleen. Bars connected with star/s differ significantly (*P < 0.05; **P < 0.01; ***P < 0.001; ****P < 0.0001). *D21: P-value = 0.23; D28: P-value = 0.55; D35: P-value = 0.17.*

No significant differences were observed in CD4+:CD8+ T cells in the spleen of birds across all treatment groups at any time point (Figure 3).

### 3.6 Effect of synbiotic vs. antibiotic supplementation on anti *C. perfringens* IgA in the bile

**FIGURE 4:**
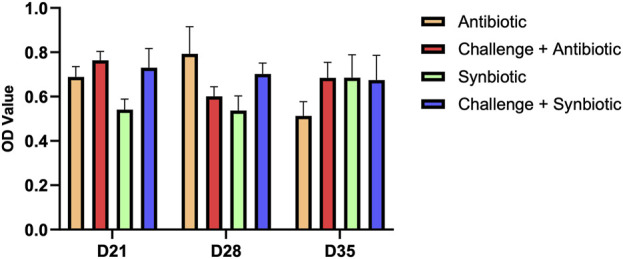
Effect of synbiotic vs. antibiotic supplementation on anti *C. perfringens* IgA production in the bile of broiler birds under an experimental NE challenge. Day-old chicks were distributed into four treatment groups. At days 21, 28, and 35, indirect ELISA was performed to measure the anti *C. perfringens* IgA in the bile. Bars connected with star/s differ significantly (*P < 0.05; **P < 0.01; ***P < 0.001; ****P < 0.0001). *D21: P-value = 0.07; D28: P-value = 0.13; D35: P-value = 0.46*.

No significant differences were observed in anti *C. perfringens* IgA levels in the bile of birds across all treatment groups at any time point (Figure 4).

### 3.7 Effect of synbiotic vs. antibiotic supplementation on expression of tight junction proteins in the jejunum and expression of cytokine genes in the cecal tonsil

**FIGURE 5:**
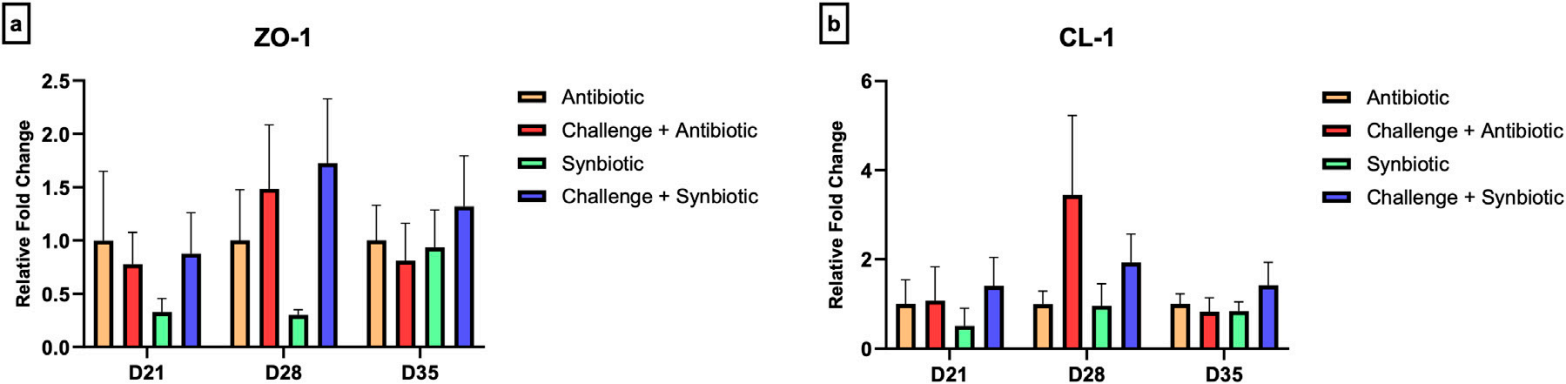
Effect of synbiotic vs. antibiotic supplementation on relative gene expression of tight junction proteins in the jejunum of broiler birds under an experimental NE challenge. Day-old chicks were distributed into four treatment groups. At days 21, 28, and 35, real-time PCR was performed to measure the relative expression of tight junction proteins in the jejunum. Bars connected with star/s differ significantly (*P < 0.05; **P < 0.01; ***P < 0.001; ****P < 0.0001). **(A)** Relative Expression of ZO-1 in the jejunum. *D21: P-value = 0.68; D28: P-value = 0.21; D35: P-value = 0.81.* **(B)** Relative Expression of CL-1 in the jejunum. *D21: P-value = 0.76; D28: P-value = 0.27; D35: P-value = 0.58.*

**FIGURE 6:**
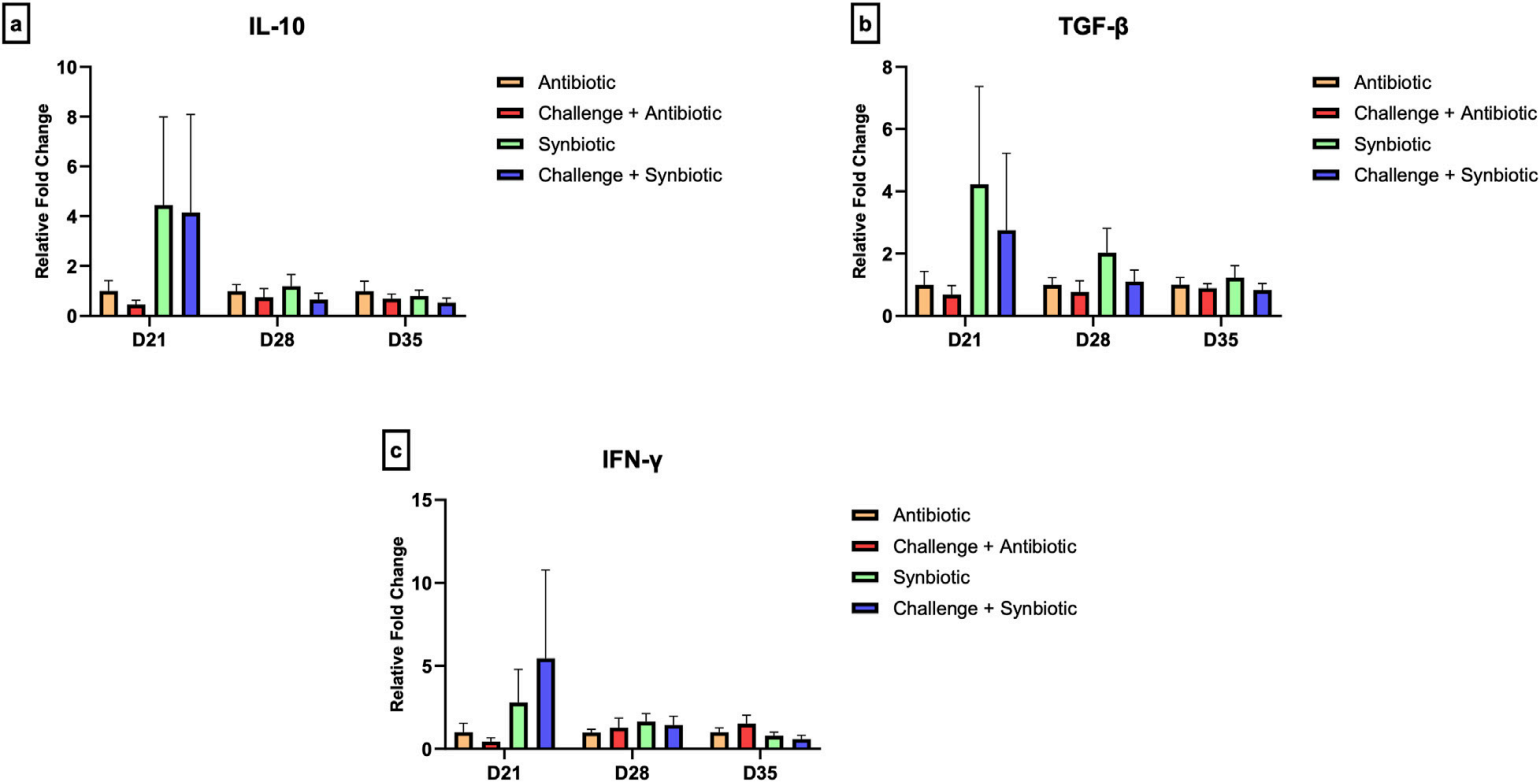
Effect of synbiotic vs. antibiotic supplementation on relative gene expression of cytokines in the cecal tonsil of broiler birds under an experimental NE challenge. Day-old chicks were distributed into four treatment groups. At days 21, 28, and 35, real-time PCR was performed to measure the relative expression of cytokines in the cecal tonsil. Bars connected with star/s differ significantly (*P < 0.05; **P < 0.01; ***P < 0.001; ****P < 0.0001). **(A)** Relative Expression of IL-10 in the jejunum. *D21: P-value = 0.61; D28: P-value = 0.68; D35: P-value = 0.65.* **(B)** Relative Expression of TGF-β in the jejunum. *D21: P-value = 0.58; D28: P-value = 0.3; D35: P-value = 0.71.* **(C)** Relative Expression of IFN-γ in the jejunum. *D21: P-value = 0.61; D28: P-value = 0.81; D35: P-value = 0.25.*

No significant differences were observed in the relative expression of tight junction proteins in the jejunum and the relative expression of cytokine genes in the CT across all treatment groups at any time point (Figures 5, 6).

## 4 Discussion

The re-emergence of NE in poultry production, following the transition to antimicrobial-free feed, has increased the need for effective antimicrobial alternatives. This study aims to assess synbiotic supplementation’s efficacy in improving broilers’ health in NE-induced experiments and compare whether its efficacy withstand the efficacy of antimicrobials.

During the first 2 weeks of age (before challenge), synbiotic and antibiotic groups demonstrated similar body weight gain, feed consumption, and feed conversion ratios. Similar results were observed when the same synbiotic was compared against the antibiotic-supplemented group, indicating that neither product provided a significant advantage over the other (Cason et al., 2023).

Birds were challenged with *E. maxima* on D14 and then with *C. perfringens* on D19, D20, and D21 to induce clinical NE, which resulted in high mortality within the challenged treatment groups. On D21 of age, challenged treatment groups supplemented with either antibiotic or synbiotic showed a decrease in body weight gain compared to their respective non-challenged treatment groups. The decrease in performance parameters following the induced NE challenge was previously reported (Akerele et al., 2022; Shah et al., 2023). In our study, the reduction in performance parameters was associated with increased intestinal permeability and high lesion scores, indicating extensive damage to the intestine. *C*. *perfringens* produces various toxins and enzymes that primarily target the cell membrane of enterocytes (Navarro et al., 2018), as well as also produces an enterotoxin (CPE) capable of binding to the claudin family, namely, claudin-3 and claudin-4. This binding increases the paracellular passage of *C*. *perfringens* through the enterocytes, exacerbating inflammation and ultimately damaging the gut barrier (Black et al., 2015).

Despite the comparable decreases in performance parameters and intestinal damage between the challenged treatment groups, the C + S treatment group exhibited a higher mortality percentage (35%) compared to the C + A treatment group, indicating that the synbiotic failed to protect against the challenge on D21. The high mortality and compromised performance in challenged treatment groups indicates that the induced NE infection was clinical in nature. Antibiotics are well-documented to target pathogenic bacteria within hours of administration (Zheng et al., 2017). Constant supplementation of antibiotics in the feed provided broilers with protection against the rapid proliferation of *C. perfringens*, whereas the synbiotic could not adapt to the rapid bacterial increment. The ratio of CD4+/CD8+ T cells is used to assess immune competency in various animals, including chickens (Char et al., 1990). According to studies, healthy individuals typically exhibit a higher percentage of CD4^+^ T cells than CD8^+^ T cells, resulting in a CD4+:CD8+ T cell ratio greater than 1. However, multiple factors such as age, breed, nutrition, and diseases, can influence CD4+:CD8+ T cells in chickens (Bridle et al., 2006; Dalgaard et al., 2010; Leshchinsky and Klasing, 2003; TA et al., 2017). At D21 of age, antibiotic supplementation resulted in decreased CD4+:CD8+ T cells compared to other treatment groups. Previous studies have reported a similar decrease in the CD4+:CD8+ T cells ratio following antibiotic supplementation in chickens (Klaudia and Alina, 2015; Lee et al., 2012). In mice, antibiotic therapy has been indicated to have a suppressive effect on Stat1 signaling, a major transcription factor related to pro-inflammatory cytokines that leads to T-cell activation, and something similar could be happening in our chicken model (Josefsdottir et al., 2017). On the other hand, no significant differences were observed in the humoral immune response, as indicated by IgA levels in the bile, between the different treatment groups. IgA is the major component of humoral immune response in the gut, and it is responsible for protecting the gut barrier against pathogenic bacteria. The lack of B cell activation, coupled with increased intestinal permeability and lesion scores, could potentially explain the high mortality and poor performance in the challenged treatment groups.

On D28 of age, the challenged treatment groups exhibited comparable levels of serum FITCd to their respective non-challenged treatment groups. A previous study highlighted that the intestinal barrier of broilers challenged with *E. maxima* on D14 and *C. perfringens* on D19, D20, and D21 showed similar serum FITCd levels on D28 of age, indicating a rapid recovery of the enterocytes following the induced-NE challenge (Akerele et al., 2022; Shah et al., 2023). All treatment groups had comparable CD4+:CD8+ ratio, IgA, cytokines (IL-10, TGF-β, and IFN-γ) and tight junction proteins (ZO-1 and CL-1) relativegene expression. However, C + A group showed comparable body weight and feed conversion ratios to their respective non-challenged treatment groups, while the C + S group failed to exhibit a similar recovery rate. The difference in recovery speed may be attributed to the antibiotic’s ability to reduce induced gut inflammation. Nevertheless, by D35 of age, all treatment groups displayed comparable performance parameters, serum FITCd levels, ratios of CD4^+^ and CD8^+^ T cells, IgA levels, and tight junction proteins and cytokine expression, indicating a possible complete recovery of the gut from the induced NE challenge.

In conclusion, both synbiotic and antibiotic supplementation supported the recovery of broiler’s performance following the induced NE challenge. However, synbiotic supplemented birds was not able to withstand the mortality caused by clinical necrotic enteritis. Therefore, supplementing synbiotic from the day of hatch can be considered a viable alternative to antibiotics for improving performance and combating the re-emergence of NE. Further study is required for improvement of the efficacy of synbiotic which might be achieved by blending additional probiotic bacteria and prebiotics. We also need to explore how mortality in chickens could be prevented by studying the in-depth immunomodulation properties of synbiotic during NE.

## Data Availability

Publicly available datasets were analyzed in this study. This data can be found here: https://www.ncbi.nlm.nih.gov and accessed with following gene accession numbers; RPS-13: NM_001001783.2, IL-10: NM_001004414.4, TGF-β: HE646744.1, IFN-G: NM_205149.2, ZO-1: XM_004934975.5, and CL-1: NM_001013611.2.

## References

[B1] Adhikari P. Kiess A. Adhikari R. Jha R. (2020). An approach to alternative strategies to control avian coccidiosis and necrotic enteritis. J. Appl. Poult. Res. 29 (2), 515–534. 10.1016/j.japr.2019.11.005

[B2] Akerele G. Al Hakeem W. G. Lourenco J. Selvaraj R. K. (2022). The effect of necrotic enteritis challenge on production performance, cecal microbiome, and cecal tonsil transcriptome in broilers. Pathogens 11 (8), 839. 10.3390/pathogens11080839 36014961 PMC9414309

[B3] Black J. D. Lopez S. Cocco E. Schwab C. L. English D. P. Santin A. D. (2015). *Clostridium perfringens* enterotoxin (CPE) and CPE-binding domain (c-CPE) for the detection and treatment of gynecologic cancers. Toxins (Basel) 7 (4), 1116–1125. 10.3390/toxins7041116 25835384 PMC4417958

[B4] Bridle B. W. Julian R. Shewen P. E. Vaillancourt J.-P. Kaushik A. K. (2006). T lymphocyte subpopulations diverge in commercially raised chickens. Can. J. Veterinary Res. 70 (3), 183–190. PMC1477934 16850940

[B5] Brynestad S. Granum P. E. (2002). *Clostridium perfringens* and foodborne infections. Int. J. Food Microbiol. 74 (3), 195–202. 10.1016/S0168-1605(01)00680-8 11981970

[B6] Cason E. E. Al Hakeem W. G. Adams D. Shanmugasundaram R. Selvaraj R. (2023). Effects of synbiotic supplementation as an antibiotic growth promoter replacement on cecal Campylobacter jejuni load in broilers challenged with C. jejuni. J. Appl. Poult. Res. 32 (2), 100315. 10.1016/j.japr.2022.100315

[B7] Char D. Sanchez P. Chen C. L. Bucy R. P. Cooper M. D. (1990). A third sublineage of avian T cells can be identified with a T cell receptor-3-specific antibody. J. Immunol. 145 (11), 3547–3555. 10.4049/jimmunol.145.11.3547 2123221

[B8] Chen Y. P. Cheng Y. F. Li X. H. Yang W. L. Wen C. Zhuang S. (2017). Effects of threonine supplementation on the growth performance, immunity, oxidative status, intestinal integrity, and barrier function of broilers at the early age. Poult. Sci. 96 (2), 405–413. 10.3382/ps/pew240 27418662

[B9] Dalgaard T. S. Norup L. R. Rubbenstroth D. Wattrang E. Juul-Madsen H. R. (2010). Flow cytometric assessment of antigen-specific proliferation in peripheral chicken T cells by CFSE dilution. Veterinary Immunol. Immunopathol. 138 (1), 85–94. 10.1016/j.vetimm.2010.07.010 20739071

[B10] Dibner J. J. Richards J. D. (2005). Antibiotic growth promoters in agriculture: history and mode of action. Poult. Sci. 84 (4), 634–643. 10.1093/ps/84.4.634 15844822

[B11] Ficken M. (1991). Necrotic enteritis. Dis. Poult., 264–267.

[B12] Helmboldt C. F. Bryant E. S. (1971). The pathology of necrotic enteritis in domestic fowl. Avian Dis. 15 (4), 775–780. 10.2307/1588866 5159549

[B13] Hutsko S. L. Meizlisch K. Wick M. Lilburn M. S. (2016). Early intestinal development and mucin transcription in the young poult with probiotic and mannan oligosaccharide prebiotic supplementation. Poult. Sci. 95 (5), 1173–1178. 10.3382/ps/pew019 26944966

[B14] Josefsdottir K. S. Baldridge M. T. Kadmon C. S. King K. Y. (2017). Antibiotics impair murine hematopoiesis by depleting the intestinal microbiota. Blood 129 (6), 729–739. 10.1182/blood-2016-03-708594 27879260 PMC5301822

[B15] Kaiser P. Rothwell L. Galyov E. E. Barrow P. A. Burnside J. Wigley P. (2000). Differential cytokine expression in avian cells in response to invasion by *Salmonella typhimurium*, Salmonella enteritidis and Salmonella gallinarum. Microbiology 146 (12), 3217–3226. 10.1099/00221287-146-12-3217 11101679

[B16] Keyburn A. L. Boyce J. D. Vaz P. Bannam T. L. Ford M. E. Parker D. (2008). NetB, a New toxin that is associated with avian necrotic enteritis caused by *Clostridium perfringens* . PLoS Pathog. 4 (2), e26. 10.1371/journal.ppat.0040026 18266469 PMC2233674

[B17] Klaudia C. Alina W. (2015). The influence of enrofloxacin, florfenicol, ceftiofur and *E. coli* LPS interaction on T and B cells subset in chicks. Veterinary Res. Commun. 39 (1), 53–60. 10.1007/s11259-015-9632-7 PMC4330464 25686865

[B18] Lee K. W. Lillehoj H. S. Lee S. H. Jang S. I. Park M. S. Bautista D. A. (2012). Effect of dietary antimicrobials on immune status in broiler chickens. Asian-Australas J. Anim. Sci. 25 (3), 382–392. 10.5713/ajas.2011.11259 25049577 PMC4092964

[B19] Leshchinsky T. V. Klasing K. C. (2003). Profile of chicken cytokines induced by lipopolysaccharide is modulated by dietary alpha-tocopheryl acetate. Poult. Sci. 82 (8), 1266–1273. 10.1093/ps/82.8.1266 12943297

[B20] Lu J. Idris U. Harmon B. Hofacre C. Maurer J. J. Lee M. D. (2003). Diversity and succession of the intestinal bacterial community of the maturing broiler chicken. Appl. Environ. Microbiol. 69 (11), 6816–6824. 10.1128/AEM.69.11.6816-6824.2003 14602645 PMC262306

[B21] Moore R. J. (2016). Necrotic enteritis predisposing factors in broiler chickens. Avian Pathol. 45 (3), 275–281. 10.1080/03079457.2016.1150587 26926926

[B22] Navarro M. A. McClane B. A. Uzal F. A. (2018). Mechanisms of action and cell death associated with *Clostridium perfringens* toxins. Toxins 10 (5), 212. 10.3390/toxins10050212 29786671 PMC5983268

[B23] NRC (1994). Nutrient requirements of poultry: 1994. Washington, DC: National Academies Press.

[B24] Oxford J. H. Selvaraj R. K. (2019). Effects of glutamine supplementation on broiler performance and intestinal immune parameters during an experimental coccidiosis infection. J. Appl. Poult. Res. 28 (4), 1279–1287. 10.3382/japr/pfz095

[B25] Porter R. E. (1998). Bacterial enteritides of poultry. Poult. Sci. 77 (8), 1159–1165. 10.1093/ps/77.8.1159 9706083

[B26] Schrezenmeir J. de Vrese M. (2001). Probiotics, prebiotics, and synbiotics—approaching a definition1,2,3. Am. J. Clin. Nutr. 73 (2), S361–S364. 10.1093/ajcn/73.2.361s 11157342

[B27] Shah B. R. Hakeem W. A. Shanmugasundaram R. Selvaraj R. K. (2023). Effect of synbiotic supplementation on production performance and severity of necrotic enteritis in broilers during an experimental necrotic enteritis challenge. Poult. Sci. 102 (10), 102959. 10.1016/j.psj.2023.102959 37619505 PMC10470215

[B28] Shanmugasundaram R. Applegate T. J. Selvaraj R. K. (2020a). Effect of Bacillus subtilis and Bacillus licheniformis probiotic supplementation on cecal Salmonella load in broilers challenged with salmonella. J. Appl. Poult. Res. 29 (4), 808–816. 10.1016/j.japr.2020.07.003

[B29] Shanmugasundaram R. Markazi A. Mortada M. Ng T. T. Applegate T. J. Bielke L. R. (2020b). Research Note: effect of synbiotic supplementation on caecal *Clostridium perfringens* load in broiler chickens with different necrotic enteritis challenge models. Poult. Sci. 99 (5), 2452–2458. 10.1016/j.psj.2019.10.081 32359580 PMC7597401

[B30] Shanmugasundaram R. Mortada M. Cosby D. E. Singh M. Applegate T. J. Syed B. (2019). Synbiotic supplementation to decrease Salmonella colonization in the intestine and carcass contamination in broiler birds. PLoS One 14 (10), e0223577. 10.1371/journal.pone.0223577 31600299 PMC6786831

[B31] Shanmugasundaram R. Selvaraj R. K. (2012a). Effect of killed whole yeast cell prebiotic supplementation on broiler performance and intestinal immune cell parameters. Poult. Sci. 91 (1), 107–111. 10.3382/ps.2011-01732 22184435

[B32] Shanmugasundaram R. Selvaraj R. K. (2012b). Regulatory T cell properties of thymic CD4+CD25+ cells in ducks. Veterinary Immunol. Immunopathol. 149 (1), 20–27. 10.1016/j.vetimm.2012.05.019 22717168

[B33] Shanmugasundaram R. Sifri M. Selvaraj R. K. (2013). Effect of yeast cell product supplementation on broiler cecal microflora species and immune responses during an experimental coccidial infection. Poult. Sci. 92 (5), 1195–1201. 10.3382/ps.2012-02991 23571328

[B34] Song D. Li A. Wang Y. Song G. Cheng J. Wang L. (2022). Effects of synbiotic on growth, digestibility, immune and antioxidant performance in broilers. Animal 16 (4), 100497. 10.1016/j.animal.2022.100497 35338905

[B35] Ta K. Ramesh G. Ushakumari S. Dhinakarraj G. Vairamuthu S. (2017). Age related changes in T cell subsets in thymus and spleen of layer chicken (*Gallus domesticus*). Int. J. Curr. Microbiol. App. Sci. 6 (1), 15–19. 10.20546/ijcmas.2017.601.002

[B36] Villagrán-de la Mora Z. Nuño K. Vázquez-Paulino O. Avalos H. Castro-Rosas J. Gómez-Aldapa C. (2019). Effect of a synbiotic mix on intestinal structural changes, and Salmonella typhimurium and Clostridium perfringens colonization in broiler chickens. Animals 9 (10), 777. 10.3390/ani9100777 31658619 PMC6826705

[B37] Zhang G. Mathis G. F. Hofacre C. L. Yaghmaee P. Holley R. A. Duranc T. D. (2010). Effect of a radiant energy-treated lysozyme antimicrobial blend on the control of clostridial necrotic enteritis in broiler chickens. Avian Dis. 54 (4), 1298–1300. 10.1637/9370-041410-ResNote.1 21313853

[B38] Zheng X. Wang X. Teng D. Mao R. Hao Y. Yang N. (2017). Mode of action of plectasin-derived peptides against gas gangrene-associated *Clostridium perfringens* type A. PLoS One 12 (9), e0185215. 10.1371/journal.pone.0185215 28934314 PMC5608353

